# Effectiveness of Rifabutin-Based Regimens in Treating Helicobacter pylori Infections

**DOI:** 10.7759/cureus.50541

**Published:** 2023-12-14

**Authors:** Jaikirat Singh Gugnani, Fnu Abhishek, Yash Agarwal, Abhiram Rao Damera, Harkamalpreet Kaur, Bayan Taleb, Rohan Mane, Ujjwal Soni, Kapil D Nayar

**Affiliations:** 1 Nephrology, Government Medical College, Amritsar, IND; 2 Internal Medicine, Government Medical College, Amritsar, IND; 3 College of Medicine, West Bengal University of Health Sciences, Kolkata, IND; 4 Internal Medicine, MediCiti Institute of Medical Sciences, Hyderabad, IND; 5 College of Medicine, Government Medical College, Amritsar, IND; 6 College of Medicine, Acibadem University, Istanbul, TUR; 7 Neurological Surgery, University of Nis, Nis, SRB; 8 General Medicine, University College of Medical Sciences, Delhi, IND; 9 College of Medicine, Sri Ramachandra Institute of Higher Education and Research, Chennai, IND

**Keywords:** drug regimens for h. pylori, h. pylori treatment challenges, rifabutin h. pylori, rifabutin triple therapy, h. pylori infection, h. pylori

## Abstract

Helicobacter pylori has been reported as a health problem worldwide, affecting a sizable portion of people. Peptic ulcers, gastric cancer, and various extra gastric conditions are associated with this bacterium. The rampant overprescribing of antibiotics has led to the emergence of H. pylori strains resistant to multiple antibiotics, causing a decline in the effectiveness of current treatments. Recently, there has been growing interest in researching alternative treatment options for H. pylori infections that do not respond to initial therapy. Rifabutin, a rifamycin derivative initially designed for tuberculosis treatment and preventing Mycobacterium avium complex infection, has gained attention as a potential rescue medication. It has shown efficacy against H. pylori and the potential to eradicate the bacterium when combined with other antibiotics. This systematic review article focuses on using rifabutin-based regimens as a treatment option after initial treatments have failed. The authors screened literature published in the last five years, between 2017 and 2022, across various search engines and closely examined relevant studies following the Preferred Reporting Items for Systematic Reviews and Meta-Analyses (PRISMA) criteria. The search covered a variety of electronic databases and focused on H. pylori gastritis, rifabutin-based treatment plans, and in vivo investigations in healthy individuals. The comprehensive review provides convincing evidence that rifabutin-based regimens are effective rescue treatments for H. pylori infections. Multiple studies in various areas consistently demonstrated high eradication rates, ranging from 70% to 90%, when rifabutin-containing regimens were used. The analysis found that only a tiny percentage of H. pylori strains (1%) were resistant to rifabutin therapy, further supporting the viability of Rifabutin as an alternative when other antibiotics failed to eradicate H. pylori. The cost of Rifabutin is a significant factor that may limit its accessibility, particularly in resource-constrained settings where H. pylori infection is common. Moreover, the potential side effects of Rifabutin, such as hematological problems, rashes, and digestive issues, need to be considered. However, these side effects are typically manageable and can be reduced by combining Rifabutin with other antibiotics. In conclusion, this systematic review provides evidence supporting the effectiveness of regimens derived from Rifabutin in eliminating H. pylori infections after initial therapy failure. Due to the observation that Rifabutin effectively eradicates resistant H. pylori infections, it can be considered a suitable choice for rescue therapy. Rifabutin-containing regimens should be reserved as fourth- or later-line therapy options, considering economic factors, the risk of microbial resistance, potential side effects, and the availability of alternative medications. Future research should focus on optimizing rifabutin-based regimens and investigating combination therapies that have better H. pylori eradication rates while also addressing the problem of resistant strains.

## Introduction and background

Helicobacter pylori peptic ulcers may be treated with one of less than ten medications, including metronidazole, amoxicillin, clarithromycin, furazolidone, levofloxacin, and tetracycline. Antimicrobial resistance may be avoided and effective eradication is achieved by combining these antibiotics with bismuth salts and proton pump inhibitors (PPIs) [1,2]. The World Health Organisation has designated the study and development of novel medicines for H. pylori species that are resistant to clarithromycin as a "high priority'' [3]. Primary antibiotic resistance is the problem caused by poor adherence to medication, which may result in treatment failure and subsequent resistance. Clarithromycin and metronidazole resistance is a worldwide crisis that must be addressed because chronic H. pylori infection is associated with gastric adenocarcinoma and localized B-cell lymphoma of the stomach (formerly known as gastric MALT lymphomas) [4,5,6,7]. H. pylori infections have been linked to numerous extragastric symptoms, such as ischemic heart disease and refractory anemia, according to recent studies [8,9]. Despite a global drop in the percentage of H. pylori infections successfully treated, antibiotic resistance, particularly to highly effective broad-spectrum antibiotics, has been rapidly increasing [6,10,11]. Tetracycline and amoxicillin have lower rates of resistance [6]. The World Health Organisation published a study in 2018 on the worldwide spread of antibiotic resistance, and they discovered that resistance to levofloxacin, metronidazole, and clarithromycin had reached 15% worldwide. However, metronidazole and clarithromycin secondary resistance rates in certain areas were from 30 to 70%. Most often owing to initial clarithromycin resistance, eradication efforts with the most frequently used first-line regimens of clarithromycin, amoxicillin, and/or metronidazole failed more than 20-30% of the time [12]. Despite the bismuth quadruple regimen (PPI, bismuth salts, and two antibiotics) being successful as a first- or second-line therapy, adverse effects have been recorded in 16-33% of instances, resulting in treatment cessation and failure [13]. With high levels of resistance in H. pylori infections, both first and second-line treatment options have shown minimal elimination of the organism, making it a particularly challenging situation with limited alternative options. Primarily, combinations of antibiotics are being used for this purpose. Rifabutin, a rifamycin derivative, has emerged as an alternative to rifampicin in treating Mycobacterium tuberculosis. Its effect in the prophylaxis for Mycobacterium avium complex in immunocompromised patients is well known [14,15]. In recent years, novel regimens incorporating Rifabutin have been extensively studied for their efficacy in eliminating H. pylori, showing promising results even after multiple treatment failures [2,15,16]. Furthermore, rifabutin resistance in vivo is rare, and the risk can be significantly reduced when Rifabutin is administered in combination with other antibiotics [15]. Previous trials have used Rifabutin in various combinations, doses, and treatment durations. However, comprehensive reviews of multiple regimens still need to be included. This review aims to determine the most effective rifabutin-based regimen by considering its benefits and potential drawbacks for treating this infection.

## Review

Methods

The systematic review was done in accordance with the Preferred Reporting Items for Systematic Reviews and Meta-Analyses (PRISMA) guidelines. A rigorous search strategy was developed to test this hypothesis and identify relevant literature. A comprehensive search of the various search engines and databases was done to include relevant literature.

A PubMed search was conducted using the Mesh terminology ''Rifabutin or N'-acetyl rifabutin or Mycobutin or ansatipine or ansamycin or LM 427, H pylori or helicobacter pylori, Therapy or therapeutic* or regimen* or protocol*, ''Rifabutin/administration and dosage''[Mesh] OR ''Rifabutin/adverse effects''[Mesh] OR ''Rifabutin/metabolism''[Mesh] OR ''Rifabutin/pharmacokinetics''[Mesh] OR ''Rifabutin/therapeutic use''[Mesh] OR ''Rifabutin/toxicity''[Mesh]. The MeSH methods are listed in Table 1.

**Table 1:** Data extraction using the MeSH strategy MeSH, Medical Subject Headings

MeSH Strategy Used	Results Obtained
''Helicobacter/genetics''[Mesh] OR ''Helicobacter/isolation and purification''[Mesh] OR ''Helicobacter/pathogenicity''[Mesh] OR ''Helicobacter/physiology''[Mesh] ) OR ( ''Helicobacter pylori/analysis''[Mesh] OR ''Helicobacter pylori/drug effects''[Mesh] OR ''Helicobacter pylori/etiology''[Mesh] OR ''Helicobacter pylori/genetics''[Mesh] OR ''Helicobacter pylori/isolation and purification''[Mesh] OR ''Helicobacter pylori/pathogenicity''[Mesh] OR ''Helicobacter pylori/physiology''[Mesh] )) AND ( ''Rifabutin/administration and dosage''[Mesh] OR ''Rifabutin/adverse effects''[Mesh] OR ''Rifabutin/analogs and derivatives''[Mesh] OR ''Rifabutin/analysis''[Mesh] OR ''Rifabutin/antagonists and inhibitors''[Mesh] OR ''Rifabutin/economics''[Mesh] OR ''Rifabutin/etiology''[Mesh] OR ''Rifabutin/immunology''[Mesh] OR ''Rifabutin/metabolism''[Mesh] OR ''Rifabutin/organization and administration''[Mesh] OR ''Rifabutin/pharmacokinetics''[Mesh] OR ''Rifabutin/pharmacology''[Mesh] OR ''Rifabutin/physiology''[Mesh] OR ''Rifabutin/statistics and numerical data''[Mesh] OR ''Rifabutin/supply and distribution''[Mesh] OR ''Rifabutin/therapeutic use''[Mesh] OR ''Rifabutin/toxicity''[Mesh] ) AND (treatment* OR regimen* OR therap* OR Clinical Protocols OR Treatment outcome OR Treatment Failure OR Disease Eradication OR Eradication OR Drug Therapy, Combination)	62
Rifabutin/administration and dosage''[Mesh] OR ''Rifabutin/adverse effects''[Mesh] OR ''Rifabutin/metabolism''[Mesh] OR ''Rifabutin/pharmacokinetics''[Mesh] OR ''Rifabutin/therapeutic use''[Mesh] OR ''Rifabutin/toxicity''[Mesh]	47
The number of titles obtained.	109

Data extracted via the PubMed search strategy is shown in Table 2.

**Table 2:** PubMed search strategy

PubMed Search Strategy	Results Obtained
Rifabutin AND peptic ulcer	7
Vonoprazan AND rifabutin AND Helicobacter	10
Helicobacter AND Rifabutin	48
The number of titles obtained.	65

**Figure 1:**
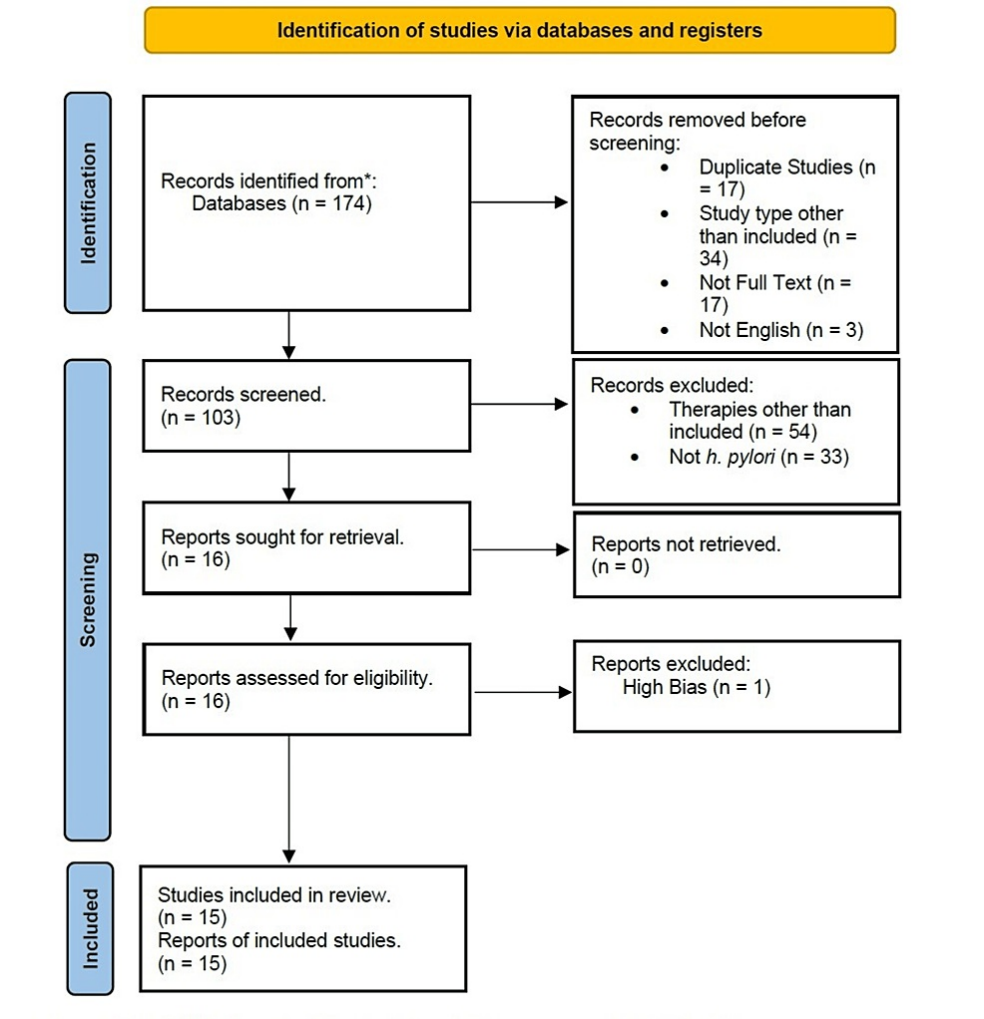
PRISMA chart PRISMA, Preferred Reporting Items for Systematic Reviews and Meta-Analyses

The authors wrote inclusion criteria, and articles were then chosen. This criterion was laid down based on various factors, including those related to H. pylori gastritis, papers discussing the research subject, publications published in English, and other specific criteria. These criteria included selecting papers with full-text articles, studies involving in vivo investigations, papers focusing on patients without comorbidities, publications published within the last five years (2016-2022), papers specifically addressing the treatment of Helicobacter gastritis, and studies examining the use of rifabutin for treating this infection, among others. We thus included randomized control trials and observational studies in this systematic review. Articles published in languages other than English, those not related to the research topic, those without full-text availability, and those based solely on in vitro studies were not included in the analysis. Patients with other gastrointestinal (GIT) diseases and comorbidities, MALTomas, and gastric adenocarcinomas were also not included. PRISMA chart is given in Figure 1.

The authors independently reviewed the research design and the possibility of bias in selection or publication. Any disagreements in the reviews of the writers were settled by consensus. A comprehensive quality assessment of the retrieved publications was conducted using the appropriate methodologies for each type of study. The results of critical appraisal using the NewCastle-Ottawa Quality Assessment Tool evaluation for case-control and cohort studies and the Cochrane Risk of Bias Tool for RCT are given in Table 3.

**Table 3:** Critical appraisal using NewCastle-Ottawa Quality Assessment Tool evaluation for case control and cohort studies and Cochrane Risk of Bias Tool for RCT RCT, randomized control trial

^Study^	^Non-Bias Percentage^
DG Ribaldone et al., 2019 [2]	100%
David Y Graham et al., 2020 [15]	66.66%
Erick A Argueta et al., 2022 [17]	77.77%
Youn I Choi et al., 2019 [18]	66.66%
Angel Cosme et al., 2017 [19]	66.66%
Kazumi Inokuchi et al., 2022 [20]	66.66%
Ira N Kalfus et al., 2020 [21]	83.33%
Chia-Jung Kuo et al., 2021 [22]	88.88%
Lee CM et al., 2022 [23]	55.55%
Miftahussurur et al., 2019 [24]	77.77%
Miftahussurur et al., 2019 [25]	77.77%
Miftahussurur et al., 2019 [26]	77.77%
Hideki Mori et al., 2016 [27]	66.66%
Saracino IM et al., 2020 [28]	44.44%
Youn I Choi et al., 2019 [29]	66.66%

Result

Fifteen studies were finalized following multiple readings of the full text of the papers and discussions among co-authors. Study characteristics are given in Table 4.

**Table 4:** Result table with study characteristics RCT, randomized control trial; OAL, omeprazole, amoxicillin, and levofloxacin; OAC, omeprazole, amoxicillin, and clarithromycin; OAM, omeprazole, amoxicillin, and metronidazole; EAR, esomeprazole, amoxicillin, and rifabutin; MRT, moxifloxacin-rifabutin triple therapy; EAC, esomeprazole, amoxicillin, and clarithromycin; EAM, esomeprazole, amoxicillin, and metronidazole; EAL, esomeprazole, amoxicillin, and levofloxacin; EBAM, esomeprazole, bismuth, amoxicillin, and metronidazole; VAR, vonoprazan, amoxicillin, rifabutin; ITT, intention to treat; PP, per protocol

Sr.no.	Number of Studies As in the References	Study Setting	Total no. of Participants	Type of Study	Mean Age	Antibiotic Regimen Used Cohort 1/Control Group/Group 1	Cohort 2/Case Group/Group 2	Mean Dose	Mean Duration	Effectiveness of Eradication in Cohort 1/Control Group	Effectiveness of Eradication in Cohort 2/Case Group	Dropouts	Resistance to Antimicrobial Drugs (%)	Year
1.	[2]	Italy	302	Cohort	57±13 years	Men (n=135). Patients received twice-daily treatments of rifabutin 150 mg, amoxicillin 1 g, and a PPI	Women (n=167). Rifabutin 150 mg, 1 g of amoxicillin, and a PPI were given to the patients twice daily	NA	14 days	In the ITT analysis, the eradication rate was 84/119 (70.6%). The remaining 19 patients had adverse reactions	In the ITT analysis, the eradication rate was 132/183 (72.1%)	5	Resistance to rifabutin was low	2019
2.	[15]	USA	455	RCT	NA	The regimen used was amoxicillin 3 g, omeprazole 120 mg, and rifabutin 150 mg, administered to 228 patients	Here amoxicillin 3 g and omeprazole 120 mg were administered to 227 individuals	NA	14 days	As per ITT analysis, the eradication rate with RHB-105 was 83.8%	As per ITT analysis, the eradication rate with the active comparator was 57.7%	NA	Resistant to rifabutin regimen=16.2%. Resistant to active comparator=42.3%	2020
3.	[17]	USA	187	Cohort study	45.2 years	101 patients on BQ regimen: PPI-tetracycline-metronidazole-bismuth (79), PPI-doxycycline- metronidazole-bismuth (18), PPI-clarithromycin-amoxicillin-metronidazole (4)	Eighty-six patients on triple regimen. PPI-clarithromycin-amoxicillin (62), PPI-clarithromycin-metronidazole (18), PPI-amoxicillin-metronidazole (4), and PPI-rifabutin-amoxicillin (2)	NA	14 days	Cohort 1: 89 (88.1%)	Cohort 2: 52 (60.5%) PP-rifabutin-amoxicillin (50%)	NA	Resistance to ≥1 antimicrobials=65.6, metronidazole=33.3, clarithromycin=30.0, levofloxacin=29.6, amoxicillin=1.1, rifabutin=0.5, tetracycline=0.5	2022
4.	[18]	Korea	31	Cohort study	58.2±10.3 years	Nine resistant to one antibiotic; however, no resistance developed to rifabutin and furazolidone	22 resistant to more than one antibiotic; however, no resistance developed to rifabutin and furazolidone	NA	NA	Cohort 1: 9 (100%)	Cohort 2: 22 (100%)	NA	Clarithromycin=71.1, levofloxacin=41.9, metronidazole=67.7, amoxicillin=22.6, rifabutin=0, furazolidone=0	2019
5.	[19]	Spain	80	Cohort study	51.92 years	Based on susceptibility, 68 patients with dual antibiotic resistance were given triple regimens: OAL (43), OAC (12), and OAM (13).	12 patients with triple antibiotic resistance were treated with OAR	NA	10 days	Cohort 1: OAL (97.6%), OAC (92.3%), and OAM (91.6%)	Cohort 2: OAR (58.3%)	NA	Dual antibiotic-resistant patients after therapy=<10% resistant. Triple antibiotic-resistant patients after treatment with rifabutin regimen=41.7%	2017
6.	[20]	Japan	69	Case control	Control: 50.3±13.9. Case group: 52.5±9.5	12 patients in historical control were given EAR therapy regimen	57 patients were given VAR therapy regimen	NA	Control, 10 days. Case Group, 7 days	Control group: as per ITT analysis, the eradication rate was 83.3%, and as per PP analysis, the eradication rate was 81.8%	Case group: as per ITT analysis, the eradication rate was 91.2%, and as per PP analysis, eradication rate was 92.7%.	18 dropouts in the case group	Resistance to 10 say rifabutin regimen=16.7%. Resistance to 7-day rifabutin regimen=8.8%	2022
7.	[21]	USA	118	RCT	46.0±10.18	RHB-105 (amoxicillin 3 g, omeprazole 120 mg, and rifabutin 150 mg) was administered to n=77 individuals	n=41. Patients were treated with placebo	NA	14 days	As per ITT analysis, 59/66, the eradication rate was 89.4%, and as per PP analysis, 56/63, eradication rate was 88.9%	As per ITT analysis, 17/33, i.e., eradication rate was 51.5%.	In the RHB-105 group: 11 dropouts, and in the placebo group: 36 dropouts	Resistance to rifabutin regimen=0%	2020
8.	[22]	Taiwan	41	Cohort	53.8 years	Antimicrobial resistance: amoxicillin (34.1%), clarithromycin (92.7%), metronidazole (65.9%), tetracycline (2.4%), levofloxacin (85.4%), and rifabutin (29.3%)	Dual antibiotic resistance to clarithromycin+levofloxacin (73.2%)	NA	10-14 days	Antimicrobial susceptibility: amoxicillin (65.9%), clarithromycin (7.3%), metronidazole (34.1%), tetracycline (97.6%), levofloxacin (14.6%), rifabutin (70.7%)	NA	NA	Resistance to rifabutin=29.3%, amoxicillin=34.1%, clarithromycin=92.7%, metronidazole=65.9%, tetracycline=2.4%, levofloxacin=85.4%	2021
9.	[23]	Korea	323	Cohort	Cohort 1: 61.7±12.6. Cohort 2: 58.0±12.1	MRT therapy regimen was administered to 71 patients following failure of the first-line clarithromycin regimen	BQ therapy regimen was administered to 252 patients following failure of the first-line clarithromycin regimen	NA	Cohort 1, 7 days. Cohort 2, 14 days	69% eradication rate in ITT analysis and 77.8% in PP analysis	82.5% eradication rate in the ITT analysis, and 89.3% in the PP analysis	NA	Resistance to MRT regimen=22.2%	2022
10.	[24]	Java Island	105	Cohort	Males range in age from 17 to 88; the mean age is (46.14±13.63). Females ranged in age from 14 to 80; the mean was 47.79±14.4 years	61/105 ( 58.1%) were sensitive to all five antibiotics examined: furazolidone, sitafloxacin, garenoxacin, rifaximin, and rifabutin. 7/105 (6.7%) were resistant to garenoxacin, and 40/105 (38.9%) were resistant to rifaximin	NA	NA	10 days	No isolates were tested for resistance to furazolidone, rifabutin, or sitafloxacin	NA	NA	Resistance to rifabutin=0, furazolidone=0, sitafloxacin=0, garenoxacin=6.7%, rifaximin=38.9% .	2019
11.	[25]	Bangladesh and Nepal	98	Cohort	NA	Nepal (n=42). Antibiotic resistance was as follows: garenoxacin 12/42 (28.6%), sitafloxacin 2/42 (4.8%), furazolidone 0/42 (0.0%), rifabutin 0/42 (0.0%), rifaximin 22/42 (52.4%)	Bangladesh (n=56). Antibiotic resistance was as follows: garenoxacin 29/56 (51.8%), sitafloxacin 1/56 (1.8%), furazolidone 0/56 (0.0%), rifabutin 0/56 (0.0%), and rifaximin 36/56 (64.3%)	NA	10 days	None of the examined strains exhibited resistance to furazolidone or rifabutin	None of the examined strains exhibited resistance to furazolidone or rifabutin	NA	Resistance in Bangladesh and Nepal to rifabutin=0, furazolidone=0	2019
12.	[26]	Dominican Republic	63	Cohort	40-49 years	Antibiotic resistance was as follows: rifaximin 52/62 (82.5%), garenoxacin 22/63 (34.9%), sitafloxacin 0/63 (0.0%), furazolidone 0/63 (0.0%), rifabutin 0/63 (0.0%)	Antibiotic resistance to rifaximin and garenoxacin is doubled (18/63) or 28.6%	NA	10 days	Rifabutin, sitafloxacin, and furazolidone are not resistant to antibiotics in the Dominican Republic. All strains were found to be responsive to rifabutin in the study, confirming the recommendation of rifabutin as a possible antibiotic to treat H. pylori infection	NA	NA	Resistance to rifabutin=0, sitafloxacin=0	2019
13.	[27]	Japan	29	Cohort	48.0±11.1 years	In the 10-day group, 12 patients received esomeprazole 20 mg q.i.d., amoxicillin 500 mg q.i.d., and rifabutin 300mg q.i.d.	The 14-day group, which included 17 patients, received esomeprazole 20 mg q.i.d., amoxicillin 500 mg q.i.d., and rifabutin 300 mg q.i.d.	NA	Cohort 1= 10 days. Cohort 2= 14 days	In the ITT analysis, the eradication rate was 10/12, i.e., 83.3%, and in the PP analysis, the eradication rate was 9/11, i.e., 81.8%. Overall eradication rate was 80%	In the ITT analysis, the eradication rate was 16/17, i.e., (94.1%), and in the PP analysis, the eradication rate was 11/12, i.e., (91.7%). Overall eradication rate was 90%	Cohort 1=1 Cohort 2=5	Resistance to rifabutin in 10 day regimen=0, in 14 day regimen=5.9%	2016
14.	[28]	Italy	1037	Single-center, open-label, single-arm prospective interventional trial	52 years	n=415. These are the patients with only one treatment regimen failure. The regimens that failed were as follows: sequential, 67/415; levofloxacin-based, 221/415; rifabutin-based, 109/415; pylera, 18/415	n= 310. These are the patients with two treatment regimen failures. The regimens that failed were as follows: sequential, 29/310; levofloxacin-based, 132/310; rifabutin-based, 129/310; pylera 20/310. n=312. These are the patients with ≥3 treatment regimen failures. The regimens that failed were as follows: sequential, 18/312; levofloxacin-based, 63/312; rifabutin-based, 216/312; pylera, 15/312	NA	10-14 days	A significant increase in the secondary resistance toward the three tested antibiotics was observed, both as rate and MIC values, in correlation with the number of therapy failures	NA	NA	Resistance to rifabutin after one therapy regimen failure=26%; Resistance to rifabutin after two therapy regimen failur=41.6%. Resistance to rifabutin after ≥3 therapy regimens failur=69.2%	2020
15.	[29]	Korea China	200	Single-center, prospective, open-label, single-arm interventional study	47.9 years	n=12 for EAC. n=13 for EAM. n=31 for EAL. EBAM, n=144	NA	NA	14 days	According to ITT analysis, the following regimens had the highest rates of eradication: EAC Group 11/12 (91.7%), EAM Group 12/13 (92.3%), EAL Group 29/31 (93.5%), and EBAM Group 137/144 (95.1%). Thus, despite numerous earlier H. pylori treatment failures, susceptibility-guided therapy proved highly efficient	NA	NA	The resistance rates for clarithromycin, metronidazole, and levofloxacin were 98.3%, 99.2%, and 93.9%, respectively, among patients who had previously received these antibiotics as part of their treatments	2019

Erick A. Argueta did research in the USA to see how new antibiotic resistance would affect efforts to eradicate H. pylori infection [17]. In a study including 101 patients, the bismuth quadruple (BQ) regimen was used. This treatment plan includes the use of bismuth, tetracycline, metronidazole, and PPIs. Eighty-six patients were given the triple regimen, which included PPIs, clarithromycin, and either amoxicillin, metronidazole, or rifabutin. The eradication rate for Cohort 2 (treated with the triple regimen) was 60.5%, whereas the eradication rate for Cohort 1 (treated with the BQ regimen) was 88.1%. The research indicated that of the antibiotics tested, metronidazole had the greatest resistance rate (33.3%), followed by clarithromycin (30%), levofloxacin (29.6%), amoxicillin (1.0%), rifabutin (0.5%), and tetracycline (0.5%). Not only that but multidrug resistance (MDR) was found in 65.6% of bacterial isolates.

Youn I Choi conducted a study in Korea to identify potential candidates for a rescue regimen in cases of antibiotic-resistant H. pylori infections [18]. Nine patients showed no elimination when just one antibiotic was used, while 22 were resistant to several. Interestingly, none of the subjects showed resistance to rifabutin or furazolidone. However, this gastric infection was completely eradicated in every patient in both cohorts, showing a 100% success rate. The study also listed the rates of resistance for several antibiotics, with clarithromycin having the highest rate at 71.1%, followed by metronidazole at 67.7%, levofloxacin at 41.9%, and amoxicillin at 22.6%. Rifabutin and furazolidone showed no signs of resistance.

In a Spanish study, susceptibility testing was used to identify treatment plans for 68 individuals with dual antibiotic resistance [19]. OAL (omeprazole, amoxicillin, levofloxacin) was administered to 43 patients, OAC (omeprazole, amoxicillin, clarithromycin) to 12 patients, and OAM (omeprazole, amoxicillin, metronidazole) to 13 patients for those with dual-resistant infections. The OAR (omeprazole, amoxicillin, rifabutin) regimen was used to treat 12 patients with triple antibiotic resistance. The study reported high eradication rates for the various regimens in both cohorts. Notably, dual antibiotic-resistant patients had a resistance rate of less than 10% following medication, indicating effective eradication. However, the resistance rate in the triple antibiotic-resistant group receiving the rifabutin-based regimen was 41.7%, indicating a lower level of effectiveness in this cohort.

David Y. Graham looked into the Rifabutin-Based Triple Therapy (RHB-105) to see if it was beneficial in treating H. pylori infection [15]. Two hundred and twenty-eight patients were given the RHB-105 regimen (amoxicillin, omeprazole, and Rifabutin) while 227 patients were given an active comparator regimen (amoxicillin and omeprazole). The study used an intention-to-treat (ITT) methodology to evaluate the eradication rates. The RHB-105 regimen had an eradication percentage of 83.8%, while the active comparator had a rate of 57.7%. According to these results, RHB-105 significantly outperformed the active comparator in eliminating H. pylori. Rifabutin showed a lower resistance rate than the active comparator, highlighting the potential benefit of utilizing it to treat infections with H. pylori.

The effects of the EAR treatment regimen (esomeprazole, amoxicillin, Rifabutin) were investigated by Kazumi Inokuchi using a historical control group of 12 patients [2]. Vonoprazan, amoxicillin, and rifabutin (VAR) were used to treat 57 different individuals. The research assessed the efficacy of two strategies for treating this infection, contrasting the ''intention-to-treat'' (ITT) approach with the ''per protocol'' (PP) approach. The case group had a greater rate of H. pylori eradication after receiving the triple therapy based on low-dose rifabutin. The 10-day regimen had a resistance rate of 16.7%, whereas the seven-day regimen had a resistance rate of 8.8%, according to the study's analysis of rifabutin regimen resistance rates.

Triple rifabutin (RHB-105) was studied by Ira N. Klafus in the USA to determine its efficacy in treating this illness [21]. Seventy-seven people were given the RHB-105 regimen (which contained amoxicillin, omeprazole, and rifabutin), and 41 people were given a placebo. In both the ITT and PP analyses, the RHB-105 regimen showed statistically significant eradication. The resistance rate to the RHB-105 regimen was 0%.

Chia Jung Kuo sought to understand better the clinical difficulties associated with multidrug-resistant infections. The trend of resistance being faced by the population was evaluated, with clarithromycin (92.7%) and levofloxacin (85.4%) having the highest resistance rates [22]. Rifabutin showed a resistance rate of 29.3%. Notably, the study disclosed neither the eradication rates attained nor the treatment regimens utilized. Instead, it focused on describing the MDR patterns and H. pylori's antibiotic susceptibility in the research population.

A study by Chang Ming Lee had 323 participants divided into two cohorts [23]. The moxifloxacin-rifabutin triple therapy (MRT) treatment regimen was administered to 71 individuals who were unsuccessful with the first-line clarithromycin regimen (Cohort 1), while 252 patients with the same condition received the BQ treatment regimen (Cohort 2). The study found that eradication rates could differ depending on the treatment plan. MRT and BQ regimens showed effectiveness as second-line treatments for peptic ulcer cases unresponsive to the first-line clarithromycin regimen.

Miftahussurur conducted research to evaluate different H. pylori infection treatment plans in Indonesian areas where metronidazole and levofloxacin resistance are common [24]. The study evaluated the susceptibility and resistance of five different antibiotics, furazolidone, sitafloxacin, garenoxacin, rifaximin, and rififabutin, to different strains of H. pylori bacteria. Among the participants, 61 out of 105 (58.1%) were responsive to each of the five antibiotics tested, indicating that these people may benefit from different eradication regimens that include these antibiotics. Notably, none of the organisms resisted sitafloxacin, furazolidone, or rifabutin. However, the study found various degrees of resistance to rifaximin and garenoxacin. Notably, 40 out of 105 subjects (38.9%) and seven out of 105 people (6.7%) were resistant to rifaximin and garenoxacin, respectively. This shows that these drugs are highly resistant in the Indonesian regions under study. The results do, however, indicate that adding furazolidone, rifabutin, and sitafloxacin to regimens can be successful in regions with great resistance to some antibiotics like levofloxacin and metronidazole.

Another study took place in the Southeast Asian countries of Bangladesh and Nepal [25]. The study assessed the regions of Nepal where there was high antibiotic resistance. Garenoxacin resistance was seen with the tested antibiotics in 12 out of 42 people (28.6%), whereas sitafloxacin resistance was seen in two out of 42 participants (4.8%). Furazolidone and Rifabutin, however, did not show any signs of resistance. Another antibiotic, rifaximin, was tested, and 22 out of 42 people (52.4%) showed resistance. A similar evaluation was carried out in Bangladesh, demonstrating the antibiotic resistance patterns in these strains. Not more than one of the 56 subjects (1.8%) demonstrated resistance to sitafloxacin, compared to 29 out of 56 patients (51.8%) who showed resistance to garenoxacin. No rifabutin or furazolidone resistance was found, similar to Nepal. Resistance to rifaximin was seen in 36 out of 56 subjects (64.3%). Importantly, none of the tested strains in either nation showed rifabutin or furazolidone resistance. This shows that despite the significant resistance seen for other regularly used antibiotics such as garenoxacin, sitafloxacin, and rifaximin, these two antibiotics could be possible candidates for effective therapy regimens against H. pylori infection in Bangladesh and Nepal.

Miftahussurur conducted a study in the Dominican Republic to find substitute antibiotics to deal with increased resistance to levofloxacin and metronidazole in treating this infection [26]. The distribution of strains of this organism in the Dominican Republic that were resistant to several antibiotics was evaluated. Rifaximin resistance was present in 52 out of 62 persons (82.5%) among the antibiotics tested, while garenoxacin resistance was seen in 22 out of 63 participants (34.9%). There was no resistance to sitafloxacin or furazolidone among the individuals. Rifabutin showed no resistance in any of the 63 participants. The study also found that 18.6% of the 63 patients exhibited double antibiotic resistance to rifaximin and garenoxacin. The study's conclusions are essential as they demonstrate how rare antibiotic resistance to rifabutin, sitafloxacin, and furazolidone is in the Dominican Republic. Therefore, these three medicines could be used to treat this infection in this area.

To assess whether triple therapy based on rifabutin was efficient as a third- and fourth-line treatments for this infection, H. Mori conducted a study in Japan [27]. The 10-day and 14-day cohorts were each given a group of subjects. Twelve patients were administered esomeprazole, amoxicillin, and rifabutin four times daily in the 10-day group. The same drugs were given to 17 individuals in the 14-day group; however, rifabutin was only given once daily. In this study, we used ITT and PP analyses to evaluate the success rate at curing H. pylori infection as our major end measure. In the ITT analysis, the eradication rate for the 10-day group was 83.3%, while in the PP analysis, it was just 81.8%. Eighty percent of patients saw improvement after 10 days of treatment. ITT analysis showed a 94.1% eradication rate in the 14-day group, and PP analysis showed a 91.7% eradication rate. The 14-day treatment plan was successful in curing 90% of the illness. No one in the 10-day group showed resistance to rifabutin, whereas 5.9% of those in the 14-day group did. Rifabutin-based triple therapy showed promise in the study as a therapeutic option when first- and second-line treatments failed to eradicate this infection.

In a sizable cohort of people suffering from this infection who had not responded to other treatment options, a trial by David et al. in Italy examined how effective this rifabutin-based rescue therapy was for treating this infection. For 14 days, all individuals received treatment with rifabutin, amoxicillin, and a PPI. ITT analysis was used to determine the primary objective: the percentage of these infections that had been eliminated completely. Male participants in the ITT study had an eradication rate of 70.6%, while female participants had an eradication rate of 72.1%. It is noted that 19 patients had adverse reactions, indicating side effects from the medication. The study stated that rifabutin resistance was minimal, but no information was given regarding its prevalence or extent. Overall, the study showed that rifabutin-based rescue therapy produced reasonably high eradication rates in patients with this infection who were challenging to treat.

The research conducted in Italy by Saracino looked into antibiotic resistance and treatment results in individuals who had failed to eliminate this infection [28]. The participants then got categorized depending on the number of treatment regimen failures they had experienced. Among the participants with only one treatment regimen failure (n=415), the failed regimens included sequential therapy in 67 patients, levofloxacin-based therapy in 221 patients, rifabutin-based therapy in 109 patients, and pylera therapy in 18 patients. The second group consisted of patients with two treatment regimen failures (n=310), and the third group comprised patients who had experienced three or more treatment regimen failures (n=312). The specific failed regimens in these groups were similar to the first group, with varying frequencies. The trial gave the recruited subjects a 10- to 14-day therapy period. According to both resistance rates and minimum inhibitory concentration (MIC) values, the study found a considerable rise in secondary resistance to the tested antibiotics, including Rifabutin. This rise in resistance was closely related to how frequently the patients' treatments failed. The study brought attention to the fact that continued treatments that were eventually not able to eliminate the organism only led to an increase in building up resistance. Notably, the resistance to rifabutin grew with each succeeding treatment failure, reaching resistance rates of 26% after one unsuccessful treatment regimen, 41.6% after two unsuccessful regimens, and 69.2% after three unsuccessful regimens or more.

YI Choi's study looked at possible rescue treatments for these infection strains that were resistant to antibiotics in China and Korea [29]. The esomeprazole, amoxicillin, and clarithromycin (EAC) group had 12 patients, the esomeprazole, amoxicillin, and metronidazole (EAM) group had 13 patients, the esomeprazole, amoxicillin, and levofloxacin (EAL) group had 31 patients, and the esomeprazole, bismuth, amoxicillin, and metronidazole (EBAM) group had 144 patients. These groups were divided based on the rescue regimens given. For a total of 14 days, the subjects underwent their rescue regimens. As per the analysis, the study discovered high eradication rates in all groups. For the EAC group, the eradication rate was 91.7%; for the EAM group, it was 92.3%; for the EAL group, it was 93.5%; and for the EBAM group, it was 95.1%. These findings show that susceptibility-guided therapy with the rescue regimens was very successful in individuals with several past H. pylori treatment failures.

Among patients who had previously taken these antibiotics as part of their treatments, resistance levels for clarithromycin, metronidazole, and levofloxacin were 93.9%, 99.2%, and 98.3%, respectively. This emphasizes the significance of modifying treatment plans in accordance with profiles of antibiotic susceptibility to increase effectiveness.

Overall, the research points to rifabutin and furazolidone as potential treatments for this infection that are resistant to antibiotics when used in rescue regimens. Research like this highlights the need to tailor antibiotic treatment plans to each patient's unique resistance profile in order to effectively eliminate H. pylori.

Finally, the study and the resources we selected shed insight into the difficulties of antibiotic resistance in the treatment of H. pylori infections. Antibiotic resistance is a major problem that threatens the success of treatment plans. Rifabutin and furazolidone are two medicines that have shown promise as effective alternatives for treating H. pylori strains that are resistant to traditional antibiotics.

These findings demonstrate the critical importance that individualized treatment regimens and susceptibility-guided therapy play in obtaining effective eradication rates. Healthcare providers may increase the likelihood of effective treatment results by choosing the most suitable antibiotics by analyzing the resistance patterns of specific patients.

Further research and ongoing surveillance of antibiotic resistance patterns in this microorganism are essential to continuously refine treatment strategies and combat the growing problem of antibiotic resistance. Additionally, developing new antibiotics and innovative treatment approaches may be necessary to ensure effective management of these infections in the future.

Discussion

This 18-month study investigated the effect of treatment failure on 187 US patients diagnosed with H. pylori infection [17]. Next-generation sequencing was applied in this study to determine H. pylori's susceptibility to different types of antibiotics. Researchers believed this procedure was quite effective because it worked in 95% of the cases and yielded results in 72 hours. Sequencing led to the administration of two separate 14-day treatment regimens to groups of 101 and 86 patients, respectively; these were the BQ regimen and the triple regimen. After this intervention, it was observed that H. pylori exhibited antibiotic resistance rates ranging from 29.6% to 33.3% to levofloxacin, clarithromycin, and metronidazole. The rifabutin-PPI-amoxicillin triple regimen was administered to two individuals. With the BQ regimen, the overall success in eliminating was 88.1%, and with the triple regimen, it was 60.5%. Rifabutin, tetracycline, and amoxicillin resistance rates varied from 0.5% to 1.1%. These rates were lower than the other antimicrobial medications used in this trial. As a result, researchers concluded that early guidance on regimens reliant on these medications led to the efficient eradication of H. pylori.

Youn I Choi et al. conducted a study on 31 patients in Korea to determine the optimal antibiotic treatment strategy for eradicating multidrug-resistant H. pylori [18]. The agar dilution method, as recommended by the Clinical and Laboratory Standards Institute (CLSI), was used to determine the lowest dilution concentration (MIC) for determining antibiotic resistance. The MIC of an antimicrobial agent is the lowest concentration at which the growth of bacterial colonies is inhibited. The CLSI guidelines were used to create the criteria for determining resistance to individual antimicrobials. In this case, the MIC for clarithromycin is higher than 1 g/mL. The resistance criteria were set at larger than 0.5 g/mL for amoxicillin, 8 mg/mL for metronidazole, 4 g/mL for tetracycline, 1 g/mL for levofloxacin antibiotics, 0.25 g/mL for rifabutin, and 4 g/mL for furazolidone. MDR is the presence of resistance to two or more antimicrobials. This traditional approach of gathering data on resistance opposes that of the study by Argueta et al. [17]. Of the 31 isolated strains, around 71.0% were resistant to more than two antibiotics. There were 13 that were resistant to two antibiotics, seven that were resistant to three, and two that were resistant to four. Only nine were immune to at least one antibiotic. The most prevalent combination of medication resistance was resistance to both clarithromycin and metronidazole (two strains, 0%). According to the in vitro results, the MIC for rifabutin ranged from 0.00098 g/mL to 0.0078 g/mL, while the MIC for furazolidone was higher. No rifabutin- or furazolidone-resistant isolates were detected, despite the fact that the concentration range on agar was lower than the CLSI-established threshold. Therefore, whether used together or individually, their regimen completely eliminates even the most resilient strains of multidrug-resistant H. pylori. To fully understand the risks associated with these drugs, however, further research is necessary.

The aim of this 12-month study by Cosmo et al. in Spain was to assess the use of giving only those antibiotics that had shown susceptibility to treat the infection [19]. This approach involved the administration of a PPI and two other antibiotics for a specific period as a new treatment option in patients with significantly high resistance to more than one drug. In this study, all participants underwent stomach biopsy procedures to obtain microorganisms for bacterial culture and E-test (BioMerieux) susceptibility testing, which included 68 people with dual antibiotic resistance to clarithromycin, levofloxacin, and metronidazole who received triple regimens (OAL/OAC/OAM). A rifabutin regimen (OAR) was administered to 12 patients who had developed antibiotic resistance to all three medicines: clarithromycin, levofloxacin, and metronidazole. Following a 10-day course of medication with strict adherence to the prescribed regimens, it was discovered that 10% of patients with dual resistance and 41.7% of patients with triple resistance still had H. pylori, which had not been completely eradicated. The utilization of rifabutin in this trial specifically targeted cases with triple resistance that had been scientifically proven, resulting in different outcomes compared to studies conducted in the USA and Korea [17,18]. Consequently, rifabutin advice was not provided at an earlier stage than in previous studies. In this study, the success of eliminating the infection was 95.5% when a combination of PPI and two antibiotics was used in those who already had resistance to more than one drug.

Another study by David Y. Graham on 455 patients with this infection in the USA aimed to assess how good the RHB-105 regimen was in eliminating this infection [15]. No treatment plans were recommended for the participant's symptoms. They were between the ages of 18 and 70 when they presented with dyspepsia that had lasted for at least two weeks. Upper endoscopy and 13C urea breath test (UBT) confirmed the diagnosis. Everyone with a favorable culture, histology, or rapid urease result was eligible for randomization. The RHB-105 treatment plan called for the use of omeprazole 120 mg, clavulanic acid 3 g, and rifabutin 150 mg. Two hundred and twenty-eight patients were given this regimen for 14 days, whereas another 227 patients were given an active comparator consisting of amoxicillin and omeprazole alone. In the ITT analysis, the RHB-105 regimen significantly outperformed the active comparator regarding the H. pylori eradication rate (83.8% vs. 57.7%). Susceptibility testing was performed for patients who experienced treatment failures; however, unlike in other studies from the USA, Korea, and Spain, the method employed in this study was not disclosed [17,18]. Based on the standard followed in this study, first-line treatment for any infection should have the highest probability of effectiveness. This is true for antimicrobial therapies. Unlike previous research, this study focused on the care of uninformed patients and concluded that the RHB-105 regimen could successfully eradicate H. pylori. This suggests that the problem of resistance to existing anti-H. pylori treatment may be handled by giving rifabutin and equivalent regimens early in the course of the illness.

A new low-dose regimen of rifabutin with vonoprazan and amoxicillin was given for seven days, and effects studies by Inokuchi et al. on 69 patients with resistant gastric infections in Japan [20]. In contrast to a study conducted in the USA, the patients in this trial had treatment failure up to the sixth therapeutic regimen [15]. According to the MIC criteria established by the CSIL and utilized in the study by Youn I Choi et al., antimicrobial susceptibility was evaluated [18]. Twelve patients served as the historical control group and were given the 10-day course of the EAR therapy regimen (esomeprazole, amoxicillin, rifabutin). However, 57 patients were treated with the VAR regimen (lansoprazole, amoxicillin, and rifabutin) for seven days. ITT analysis showed that the eradication rate for VAR therapy was 91.2%, while it was only 83.3% for the EAR treatment group.

The median rifabutin MIC for all strains was 0.00 0.01 g/mL. When comparing the two groups' safety profiles, those in the seven-day VAR group fared worst, with 31.6% (18/57) reporting side effects and two people dropping out of the study due to adverse reactions. Both patients stopped taking the medication on day four; one owing to stomach pain and the other due to headache and conjunctival hyperemia. There were no serious adverse events, and the symptoms of each patient diminished when the medicine was used up. Unlike previous studies conducted in the USA, South Korea, Japan, and Spain, this one really assessed potential risks. In this study, a seven-day low-dose rifabutin-based VAR treatment was shown to be a reliable and secure way to get rid of H. pylori. With the use of early advice for rifabutin regimens, this infection might be treated and symptoms reduced sooner rather than later.

Isolates of H. pylori from individuals with refractory infections showed extremely high levels of antibiotic resistance, which greatly hampered effective therapy. The study discovered that resistance rates were quite high for well-known drugs such as metronidazole, levofloxacin, amoxicillin, and clarithromycin. Many people had dual resistance to clarithromycin and levofloxacin. The results highlighted the urgent need for alternative H. pylori treatment methods [21]. When clarithromycin failed to completely cure H. pylori infection, the efficacy of BQ treatment and MRT was examined. The results showed that BQ treatment, compared to MRT therapy, was more effective in curing the infection in patients with peptic ulcers. MRT should seldom be considered when BQ therapy is not viable [22].

In conclusion, the rising likelihood of antibiotic resistance among patients with refractory H. pylori infection highlights the urgent need for novel treatment modalities. According to the trial's findings, BQ therapy is superior to MRT as a second-line therapy for this condition. Even yet, further investigation is necessary to identify complementary treatments for those with resistant strains of H. pylori [21,22].

This retrospective research was carried out by Chang Ming Lee et al. [23]. Due to limited patient compliance and a convoluted dose strategy, finding a suitable third-line treatment when BQ therapy fails is difficult. Several Korean regimens failed to completely eradicate the illness. Therefore, the researchers looked at how well BQ treatment for peptic ulcers compared to the second-line MRT combination for curing the infection. Between January 2013 and December 2019, 665 H. pylori patients who had failed to respond to first-line clarithromycin were continually recruited at Gyeongsang National University Hospital. A total of 665 participants were included in the trial, with 71 receiving BQ and 252 receiving MRT. In order to conduct the peptic ulcer subgroup analysis, 132 patients from the BQ group and 51 patients from the MRT group were randomly selected. The study included 323 patients who met the inclusion criteria. Using an age and gender matching mechanism, 45 patients were examined, 15 from each group. Sixty-nine point zero percent of the cases were eradicated in the MRT group. These rates were much lower than the eradication rate seen in the BQ group, indicating that the treatment was far more successful. Analysis of patients who suffered from peptic ulcers revealed that those in the BQ group fared much better than those in the MRT group. Thirteen of the 14 patients who did not improve with MRT were cured after switching to a BQ regimen in the third line of treatment. BQ was effectively eradicated at a 90% rate after MRT had failed. From these findings, the researchers drew the conclusion that MRT treatment is not as successful as BQ therapy and should only be explored in cases when BQ is not possible.

Miftahussurur et al. did research to determine effective therapy for this illness in areas of Indonesia where metronidazole and levofloxacin resistance are prevalent [24]. Clarithromycin and metronidazole resistance are serious problems in Indonesia. Resistance to levofloxacin and bismuth therapy is rising, which has resulted in a rise in the use of other antibiotic treatments. The dilution test was performed to determine how resistant H. pylori was to five different antibiotics. Additionally, next-generation sequencing was employed to identify mutations associated with antibiotic resistance. After being acquired from 1039 adult dyspeptic sufferers, 106 strains were tested, none showing furazolidone resistance. Furthermore, all the tested strains showed sensitivity to both rifabutin and sitafloxacin. In contrast, rifaximin and garenoxacin exhibited high resistance rates. Notably, garenoxacin-sensitive organisms have been found in places with high levels of clarithromycin resistance. However, rifaximin might not be a good antibiotic choice due to the prevalence of clarithromycin resistance. Finally, it was seen that rifabutin-based therapies were effective in eliminating this infection compared to the conventional protocols. Sitafloxacin should be explicitly considered to eradicate levofloxacin-resistant strains, while garenoxacin should be avoided. In the study conducted by Miftahussurur and colleagues, the researchers looked at successful treatment techniques for H. pylori infections in Bangladesh and Nepal, two countries where there was a considerable amount of resistance to the primary medicines [25]. Patients with adult dyspepsia who were undergoing endoscopies at the Tribhuvan University Teaching Hospital (TUTH) were included in this study. Patients came from both Nepal and Bangladesh. After homogenizing the antral biopsy tissues, 42 strains were successfully recovered from Nepal (146 patients), while 56 strains were successfully recovered from Bangladesh. These strains were then cultivated at 37°C for up to 10 days. Subcultures of H. pylori were grown in the same microaerophilic conditions on antibiotic-free Mueller-Hinton II agar medium with 7% horse blood. Brucella broth with 10% dimethyl sulfoxide and 10% horse serum was used to keep the H. pylori solution at an ideal temperature of 80°C. Two-fold agar dilution was employed to determine antibiotic susceptibility. The five antibiotics examined were rifaximin, sitafloxacin, furazolidone, garenoxacin, and rifabutin.

Rifabutin and furazolidone were not resistant; however, sitafloxacin susceptibility was very high. In contrast, rifaximin was highly resistant. Garenoxacin resistance is also more prevalent in Bangladesh than in Nepal because of its association with levofloxacin resistance. As a remedy for this infection, Rifabutin should be taken cautiously, according to the authors, because of the drug's interaction with Bangladesh's widespread tuberculosis. The high susceptibility to furazolidone and sitafloxacin increased the prospects for their future use in Bangladesh and Nepal.

In a study, Muhammad Miftahussurur et al. sought to identify alternative H. pylori antibiotics for the Dominican Republic to mitigate resistance, which is widespread throughout the entire nation [26]. The MICs of five different antibiotics were determined using a two-fold agar dilution method against a panel of 63 different strains from the Dominican Republic. We employed next-generation sequencing to analyze genomic changes associated with antibiotic resistance. Rifabutin, furazolidone, and sitafloxacin were active against all 63 strains. On the other hand, resistance to rifaximin and garenoxacin was widespread (82.5% and 34.9%, respectively). Garenoxacin resistance (8/9, 88.9%, OR=45.33, P=0.002) and dual resistance to antibiotics (7/9, 77.8%, OR=31.5, P=0.009) were more likely in individuals older than 60 years old (P=0.004, r=0.357). More than three changes in rpoB were present in most rifaximin-resistant strains, with the most common new variants being S352L, I2726L, and V2465A. A strong correlation between the vacA genotype and rifaximin resistance was found (P=0.042). The lowest inhibitory concentration was significantly higher among the 23 levofloxacin-resistant organisms (39.1%, 9/23) and was positively correlated with resistance (P=0.001, r=0.84). Additionally, the vast majority of these organisms were resistant to garenoxacin (82.6%, 19/23, P=0.001). In a study with 10 strains, 16 (or 84.2% of the total) exhibited gyrA mutations at D91 and N87, making them resistant to garenoxacin.

The study exhibited several limitations, outlined as follows: first, due to the limited sample size and the exclusive focus on samples collected solely from the capital of the Dominican Republic, the generalizability of the results to the rest of Latin America was compromised. Second, the study examined only a subset of the numerous H. pylori genes that could potentially be associated with antibiotic resistance, thus offering a limited understanding of the topic. Third, the resistance rates of the five different antibiotics were solely estimated through in vitro experimentation. Alternatives to levofloxacin and metronidazole that could be incorporated into the eradication strategy include sitafloxacin, rifabutin, and furazolidone. Such regions include the Dominican Republic, which has a high rate of resistance to these medications.

The purpose of the prospective randomized investigation by Hideki Mori et al. is to determine whether rifabutin, used as part of a triple therapy regimen, is effective as a third- or fourth-line rescue medication [27]. Participants in the trial were those who had not improved after receiving both primary and secondary eradication treatments. A stomach biopsy was carried out to ascertain whether the rpoB mutation was present. For 10 or 14 days, patients took eradication medication with rifabutin (300 mg once daily), amoxicillin (500 mg four times daily), and esomeprazole (20 mg four times daily). Eighty percent of the sample reported that they did not take their medication as prescribed. A 13C UBT or a stool antigen test was used to establish if H. pylori had been successfully eradicated from the body after therapy had concluded. There were a total of 22 people who took part in the experiment, 12 of whom were placed in the 10-day group and 17 in the 14-day group. In the 14-day group, the success rate of eradicating the infection was 94.1% while in the 10-day group it was 81.8% (depending on the technique of analysis). Additionally, between 8% and fewer than 30% of patients in the two cohorts stopped taking their prescriptions as a result of side effects including diarrhea.

However, more patients in the 10-day group finished the fourth eradication medication, suggesting that the 14-day group's success rate may have been overestimated. In addition, there was no reliable way to assess eradication rates, rpoB mutations, or rifabutin MICs. Finally, we can say that both the 10-day and 14-day rescue regimens worked, with the latter reaching an eradication rate of over 90%. However, when therapeutic tolerance is taken into account, it is possible that a 10-day course of treatment is all that is necessary for complete eradication.

A few issues with this study should be noted. The self-reported approach for assessing therapy compliance was the first. This approach may be limited by its dubious precision and subpar assessment accuracy. Second, there needs to be more resistance data due to the exclusive reliance on clinical assessment without including susceptibility testing. As a result, the reasons for earlier treatment failures in these patients (lack of drug adherence or a rise in antibiotic resistance) are unknown. Furthermore, the study population exhibited a lack of complete homogeneity due to various characteristics of the study design.

In summary, our study demonstrated that, when repeated attempts to eradicate H. pylori using standard antibiotics failed, rifabutin-based rescue therapy is a practical and secure option. A sizable group of people who were very difficult to treat participated in the trial. In addition, Saracino et al. carried out a retrospective analysis to examine how antibiotic resistance emerged, how therapy progressed, and how MIC values changed over time in patients who had been unable to eradicate H. pylori with at least one medication [28]. The effectiveness of levofloxacin, metronidazole, and clarithromycin against H. pylori bacteria was examined in a study including individuals who had UGIE after prior medication. Pylera® or a susceptibility-guided treatment plan was provided to the patients.

Antibiotic susceptibility data was provided for 1037 of the 1223 patients who tested positive for H. pylori. Antibiotic resistance was found to be at substantially higher rates, MIC values were much lower than expected, and the number of failed treatment efforts was much higher than expected. Except for when sequential therapy is utilized as a third-line therapy, the eradication rates of antibiogram-tailored drugs remained unchanged. It was found that when Pylera® therapy was utilized as a last resort, it resulted in cure rates comparable to those of other culture-guided treatments. Unfortunately, the Pylera® regimen is difficult to administer and more frequently leads to adverse effects than alternative treatments. Up to 14 pills taken four times over the course may be necessary, and more than 30% of patients experience side effects, which increases the likelihood that therapy will be stopped early.

In conclusion, the number of therapeutic failures was inversely linked to the rate and MIC values of secondary resistance to the three tested antibiotics. The eradication rates from Pylera® therapy were comparable to those from treatments tailored to a particular culture. Rifabutin, furazolidone, and other antibacterial medications were tested for their antimicrobial efficacy in a second prospective trial by Youn I Choi et al. with 200 participants to investigate their potential efficacy as therapies for multidrug-resistant H. pylori along with H. pylori strains, which are resistant to other antibiotics [29]. After conducting a retrospective analysis of the information available at the Helicobacter Registry of two famous medical centers, along with four reference strains, a total of 31 single- or multidrug-resistant H. pylori bacteria were selected for testing. The strains were subjected to the broth microdilution technique to evaluate their resistance to a number of different antibiotics.

No rifabutin- or furazolidone-resistant H. pylori isolates were discovered among the 31 antibiotic-resistant strains. Only one of the strains tested positive for tetracycline resistance. Rifabutin and furazolidone are more effective at curing the infection than amoxicillin and levofloxacin, which have resistance rates of 22.6% and 1.9%, respectively. Only 3.2% of H. pylori bacteria were resistant to the antibiotic tetracycline. Since the eradication rate was not demonstrated by treating actual patients, it is possible that the in vitro results do not reflect what happens in vivo. It is also important to remember that there may be a disparity between the results of the study and the actual prevalence of antibiotic-resistant H. pylori in Korea due to selection bias. Clinicians should exercise caution before extrapolating the findings to the general public due to ethical considerations, and the research's lack of information on antibiotic resistance in H. pylori is another drawback.

In conclusion, rifabutin, furazolidone, and tetracycline can be used to eliminate this infection successfully. They can be used as solitary treatment options or together as a complete regimen in regions where species of this organism have developed very high rates of resistance. However, further research and careful consideration are warranted before implementing these findings on a broader scale. As H. pylori becomes increasingly resistant to medications, new approaches are required to treat this persistent infection.

The review of key data presented in this research sheds light on the challenges posed by antibiotic-resistant H. pylori and the need to develop new therapeutic techniques. In view of the proliferation of multidrug-resistant strains of H. pylori, the development of new drugs and treatment methods is essential for the successful elimination of this pathogen. Rifabutin, furazolidone, and sitafloxacin have shown promise as potential therapeutic options, especially in regions where resistance to conventional antibiotics is prevalent.

Healthcare providers must keep abreast of the most recent findings on H. pylori resistance patterns and treatment choices in order to give patients with H. pylori infection effective and individualized therapeutic interventions. Furthermore, larger-scale studies with a more representative sample of the general population are required to demonstrate the efficacy and safety of these supplementary drugs.

In conclusion, combating H. pylori infection and the ensuing rise in antibiotic resistance requires an all-encompassing strategy that includes continual monitoring, the cautious use of drugs, and the exploration of potential innovative treatments. The medical community has to make considerable efforts worldwide to improve H. pylori infection treatment and reduce the prevalence of related illnesses.

Limitations

There were no in vitro clinical trials included in this systematic review. We might have overlooked a few crucial publications because we could not obtain a few articles as the complete text was unavailable, and these were not included in our study.

## Conclusions

In conclusion, therapies that can successfully eliminate this infection in the first attempt must be considered the gold standard when prescribing medications. However, the rising global resistance to antibiotics like amoxicillin, metronidazole, clarithromycin, tetracycline, and levofloxacin raises concerns about their efficacy. Due to this, there is an increased need for developing efficient rescue regimens that can successfully eradicate this organism and prevent treatment failure. Our systematic review provides evidence showing that rifabutin regimens can be used to eliminate this infection successfully. Nearly all studies have shown that rifabutin-based regimens exhibited the lowest resistance rates compared to other drugs included in the therapy. It is noteworthy that rifabutin therapy has shown efficacy even in individuals with initial resistance to levofloxacin, clarithromycin, and metronidazole. Without relying on bacterial culture, it may be prudent to consider the empirical use of rifabutin as ''rescue therapy" in circumstances where these antibiotics have failed to work. Therefore, rifabutin-containing regimens should only be used as a fourth or subsequent therapy if early eradication regimens using other first-line antibiotics such as metronidazole, amoxicillin, clarithromycin, tetracycline, and levofloxacin fail. The microbial resistance, possible adverse effects, and the availability and efficacy of alternative drugs should all be carefully considered before choosing rifabutin as a first-line therapy choice for H. pylori infection.
