# Correction: Effectiveness of Rifabutin-Based Regimens in Treating Helicobacter pylori Infections

**DOI:** 10.7759/cureus.c158

**Published:** 2024-02-02

**Authors:** Jaikirat Singh Gugnani, Fnu Abhishek, Yash Agarwal, Abhiram Rao Damera, Harkamalpreet Kaur, Bayan Taleb, Rohan Mane, Ujjwal Soni, Kapil D Nayar

**Affiliations:** 1 Nephrology, Government Medical College, Amritsar, IND; 2 Internal Medicine, Government Medical College, Amritsar, IND; 3 College of Medicine, West Bengal University of Health Sciences, Kolkata, IND; 4 Internal Medicine, MediCiti Institute of Medical Sciences, Hyderabad, IND; 5 College of Medicine, Government Medical College, Amritsar, IND; 6 College of Medicine, Acibadem University, Istanbul, TUR; 7 Neurological Surgery, University of Nis, Nis, SRB; 8 General Medicine, University College of Medical Sciences, Delhi, IND; 9 College of Medicine, Sri Ramachandra Institute of Higher Education and Research, Chennai, IND

This article has been corrected. The article originally stated that the resistance rate to the RHB-105 regimen was 10.6% in the Klafus et al study, which could affect the treatment's efficacy. This was incorrect as it was conflated with the treatment failure rate. The resistance rate has been corrected to 0% and the statement regarding the treatment's efficacy has been removed.

